# Iron–sulfur clusters biogenesis by the SUF machinery: close to the molecular mechanism understanding

**DOI:** 10.1007/s00775-017-1527-3

**Published:** 2017-12-26

**Authors:** J. Pérard, Sandrine Ollagnier de Choudens

**Affiliations:** 1 Laboratoire de Chimie et Biologie des Métaux, Biocat, Université Grenoble Alpes, Grenoble, France; 2 Laboratoire de Chimie et Biologie des Métaux, CNRS, BioCat, UMR 5249, Grenoble, France; 3 CEA-Grenoble, DRF/BIG/CBM, Grenoble, France

**Keywords:** Biosynthesis, Iron–sulfur cluster, Metallocenter assembly, Mechanism, SUF

## Abstract

Iron–sulfur clusters (Fe–S) are amongst the most ancient and versatile inorganic cofactors in nature which are used by proteins for fundamental biological processes. Multiprotein machineries (NIF, ISC, SUF) exist for Fe–S cluster biogenesis which are mainly conserved from bacteria to human. SUF system (*sufABCDSE* operon) plays a general role in many bacteria under conditions of iron limitation or oxidative stress. In this mini-review, we will summarize the current understanding of the molecular mechanism of Fe–S biogenesis by SUF. The advances in our understanding of the molecular aspects of SUF originate from biochemical, biophysical and recent structural studies. Combined with recent in vivo experiments, the understanding of the Fe–S biogenesis mechanism considerably moved forward.

## Introduction

Iron–sulfur clusters (Fe–S) are amongst the most ancient and versatile inorganic cofactors in nature. They are used by proteins for fundamental biological processes such as nitrogen fixation, photosynthesis, respiration, DNA repair [1–4]. The most common types of Fe–S are the 2Fe–2S and the cubane 4Fe–4S clusters that contain either ferrous (Fe^2+^) or ferric (Fe^3+^) iron and sulfide (S^2−^) [2]. In most cases, thiolate from cysteine coordinate iron ions of the cluster although there are increasing examples of nitrogen coordination—provided by histidine or arginine residues—and oxygen coordination—from aspartate or tyrosine. Examples of coordination by exogenous ligands, such as water molecules, enzyme substrates or cofactors have also been observed [2]. Because of the toxicity of free iron and sulfur, the biogenesis of Fe–S cofactors must be tightly regulated. Multiprotein machineries exist for Fe–S cluster biogenesis which are mainly conserved from bacteria to human, although elaborate systems have diverged through evolution.

Three distinct types of biosynthetic machinery have emerged from bacteria, archaea and eukaryotic organelles, based on biochemical evidence and organization of genes in bacterial operon. Whereas the NIF system plays specialized roles in the maturation of Fe–S proteins in nitrogen fixing organisms such as *A. vinelandii* [5, 6], the ISC machinery is the primary system for general Fe–S cluster biosynthesis in bacteria [7]. Moreover, along with additional components, the ISC system constitutes the eukaryotic mitochondrial machinery for Fe–S cluster biogenesis. Components in eukaryotes were discovered by a variety of genetic screens performed on *Saccharomyces cerevisiae* based on Fe homeostasis, amino acid biosynthesis, ribosome biosynthesis and DNA repair [8]. The third bacterial assembly system, termed SUF, plays a similar general role as ISC in many bacteria, but is operative only under conditions of iron limitation or oxidative stress [9]. Not surprisingly, the bacterial SUF system also forms the basis of the Fe–S cluster biogenesis machinery in plant chloroplasts, an O_2_-producing organelle that is most likely inherited from the cyanobacterial ancestor of plastids [10, 11]. The SUF system also appears to be the sole system for Fe–S cluster biogenesis in archaea and cyanobacteria, as well as many Gram-positive, pathogenic and thermophilic bacteria. Genomic analyses revealed that the number and type of operons coding for these systems vary from one microorganism to another. Some contain all systems, others two or only one, and some only contain a part of one system [9, 12, 13].

**Fig. 1:**
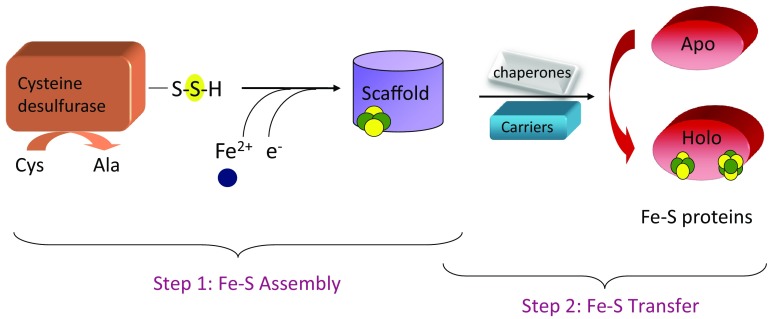
Simplified Fe–S assembly mechanism

For all systems, the basic process of Fe–S biogenesis requires donation of iron (ferric or ferrous) and sulfide as bridging ligand for iron ions. Sulfide is provided by cysteine desulfurase enzyme that uses l-cysteine as stable and safe source of sulfur, whereas origin of iron is still unclear (Fig. 1). The two components, iron and sulfide, first combine on a protein that serves as a “scaffold” for cluster assembly (Step 1, Fig. 1). Due to the lability of the scaffold bound-cluster it can be transferred to appropriate apoform of metalloprotein either directly or using a series of carriers proteins that mediate trafficking and targeting of the mature Fe–S proteins (Step 2, Fig. 1).

### Fe–S biogenesis and health

In humans, a number of genetic diseases are associated with dysfunction of the ISC system, showing the importance to study at a molecular level Fe-S cluster biogenesis process to better arrest these diseases [14, 15]. The SUF system displays also an important scientific interest in health. Indeed, the SUF machinery is not conserved in humans and it is the only Fe–S biogenesis pathway in some pathogens such as *Staphylococcus aureus* (SufSBCDUTA, and Nfu) [16, 17], *Mycobacterium tuberculosis* (SufRBDCSUT) [18], parasites *Plasmodium* (SufABCDSE) and *Toxoplasma*, making SUF an attractive pathogen-specific drug target. Recently, it was demonstrated that d-cycloserine could inhibit in vitro the cysteine desulfurase activity of *P. falciparum* SufSE (IC_50_ of 20 µM) [19]. d-Cycloserine binds to the PLP cofactor and forms a 3-hydroxyisoxazole-pyridoxamine adduct with PLP causing inhibition of the enzyme. d-Cycloserine is in clinical use as a second line drug against *M. tuberculosis* [20] and was shown to inhibit the blood stage growth of *P. falciparum* [19]. Although it was not conclusively shown that the growth inhibitory effect of d-cycloserine is due to SufS inhibition (it may inhibit other PLP enzymes) it is a promising start to identify drugs that target Suf function. Recent investigations in *S. aureus* showed that SUF system is the target system for a polycyclic molecule named molecule 882 [21]. In particular, when SuB, SufC and SufD are pulldown with molecule 882, a direct interaction between molecule 882 and SufC is observed (KD 2 µM). In agreement with this result, a strain deficient in the maturation of Fe–S biogenesis (ΔsufT, ΔnfU) displays an increased sensitivity to molecule 882 than the wild-type. All these studies prove that SUF system is a good target for an antibiotherapy and may guide the development of new antimicrobials.

## The SUF biogenesis system

**Fig. 2:**
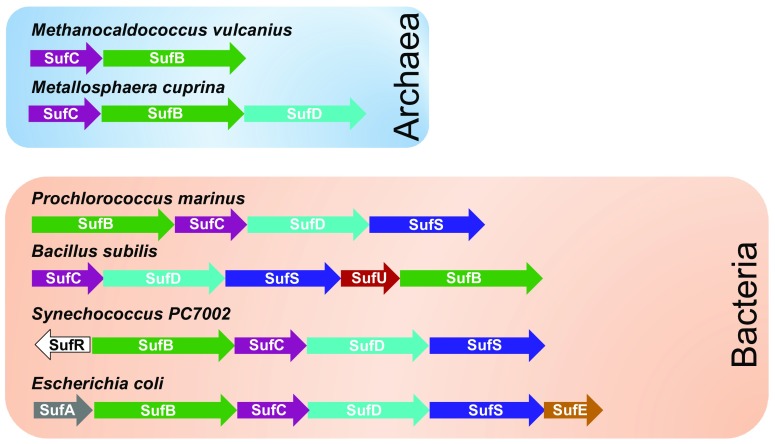
Evolution of *suf* operon. Selected examples of *suf* operons among Archae and bacteria. Genes for *sufA*, *sufB*, *sufC*, *sufD*, *sufS* and *sufU* are color-coded to reflect their homology in different organisms. Adapted from [29]

The SUF system is the most ancient of the currently identified system of biogenesis [9]. As mentioned before, in some organisms, the SUF system is the only system present, and therefore, is essential for viability. In others, SUF operates in parallel with ISC and NIF [22, 23]. Lack of a functional *suf* operon is neutral for *E. coli* under normal growth conditions [9, 12, 24]. In contrast, under oxidative stress, deletion of the *suf* genes made *E. coli* unable to produce functional forms of enzymes containing oxygen-labile Fe–S clusters [12]. The same observation was obtained when cells were exposed to 2,2′-dipyridyl, an iron chelator [12]. These observations led to the conclusion that *suf* operon is functional under oxidative stress and iron limitation. Further genetic analyses demonstrated that *suf* operon operates under stresses owing to regulators such as apo-IscR (oxidative stress and iron deprivation), Fur/RhyB (iron limitation) and OxyR (oxidative stress) [25–28]. The *suf* operon contains two (SufB, SufC) to more than six genes (SufA, SufB, SufC, SufD, SufS, SufE, SufU) organized as single polycistronic transcriptional units, showing that the role of SUF has evolved through evolution (Fig. 2). A recent phylogenetic analysis of the SUF pathway suggests that diversification into oxygen-containing environments disrupted iron and sulfur metabolism and was a main driving force in the acquisition of additional (more) SUF proteins by the SufB–SufC core [29]. Thus, there would have been an evolutionary trajectory in which *suf* grew in complexity from an operon encoding only *sufB*–*sufC* through the sequential recruitment of other genes such as *sufD*, *sufS* and *sufE*.

The SUF system has been the subject of in depth biochemical, genetic and regulatory studies, especially in *E*. *coli* and *Erwinia chrysanthemi* [22, 30–32]. From that we know that SufB, SufC, and SufD can interact with each other forming SufB_2_C_2_, SufC_2_D_2_ and SufBC_2_D complexes; SufS interacts with SufE forming a 1:1 complex and similarly SufS interacts with SufU. Finally, SufSE complex interacts to SufBC_2_D complex.

## Biochemistry of Suf proteins

### SufB

**Fig. 3:**
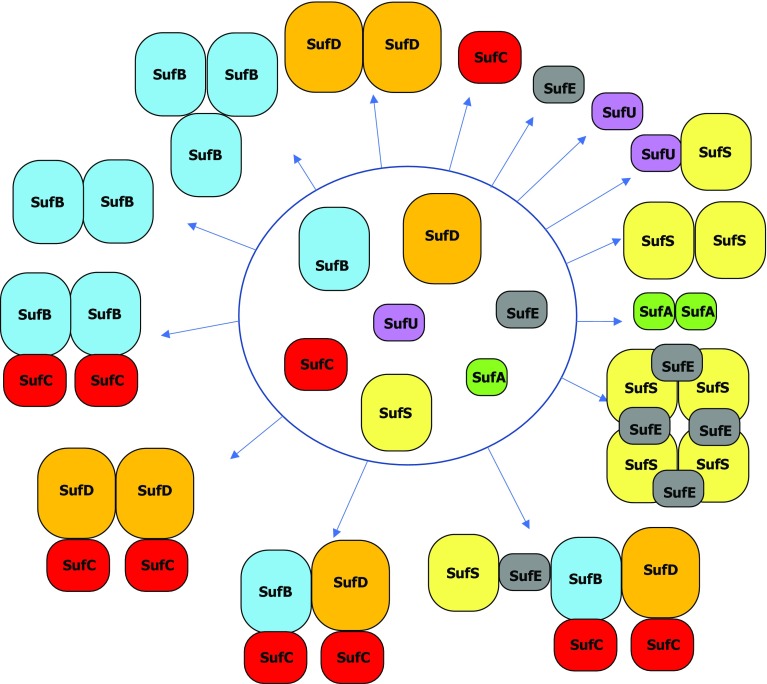
Possible interaction of Suf proteins. Interactions between Suf proteins that were identified by biochemical and biophysical studies

SufB is the scaffold protein of the system, an essential player in the process. SufB is a difficult protein to manipulate in vitro as it tends to be insoluble and it exists under different oligomerization states (Fig. 3). This likely explains that no structure of SufB is available. As a scaffold protein, SufB is able to assemble transiently a Fe–S cluster even though its nature is not clearly known. Previous studies have established that *E. coli* SufB assembles a 4Fe–4S cluster after in vitro reconstitution [33, 34]. Both 4Fe–4S and linear 3Fe–4S clusters were observed on purified His–SufB after in vivo co-expression with *sufCDSE* genes [35]. However, we discovered that SufB can stabilize a 2Fe–2S cluster after anaerobic incubation of apo-SufB with a threefold molar excess of ferric iron and sulfide and purification onto an anion exchange column [36]. Recently, in vivo experiments show that SufBC_2_D complex after an early step purification displays a typical 2Fe–2S UV-visible spectrum, reinforcing the idea that SufB might be a 2Fe–2S protein rather than a 4Fe–4S protein [37]. Interestingly, SufB 2Fe–2S cluster is more stable and resistant to H_2_O_2_, O_2_ and iron chelator than the 2Fe–2S of IscU in agreement with its function under oxidative conditions [36]. Both 2Fe–2S and 4Fe–4S holo-forms of SufB are competent for transfer for intact cluster to diverse proteins such as SufA, ferredoxin (Fdx) and aconitase [38–40]. The N terminus of SufB from *E. coli* and its close relatives contains a putative Fe–S cluster motif (CXXCXXXC) that was proposed early to be the site of Fe–S cluster assembly [33]. However, the cysteine triple mutant can still assemble a Fe–S cluster in vitro after chemical reconstitution suggesting that these cysteine are not cluster ligands (Layer et al. unpublished results). Recently, cysteines of this motif were unambiguously excluded as ligands [41]. Among the invariant cysteine residues in SufB, Cys405 (*E. coli*) is proposed to be one of the Fe–S ligand from structural studies [37] and recent in vivo experiments [41] (see below). Residues Glu434, His433 and/or Glu432 are proposed to be the other Fe–S ligands [41]. As a scaffold, SufB is able to interact with the cysteine desulfurase SufSE complex through SufE protein. The interaction between SufB and SufE occurs only if SufC is present in agreement with the existence of SufB_2_C_2_ and SufBC_2_D physiological complexes (Fig. 3). When SufB (within SufBC_2_D complex) is incubated with SufSE and l-cysteine and without reducing agent, up to seven sulfur atoms can accumulate on SufB [33]. Recently, two cysteine residues of SufB, which are strictly conserved cysteine residues, were identify as good sulfur acceptor sites from SufE: Cys254 and Cys405 [41]. Cys254A mutation abolishes sulfur accumulation while and Cys405A mutation strongly diminishes sulfur binding. Interestingly, Cys254 residue is critical for the stimulation of the cysteine desulfurase activity of SufSE by SufBC_2_D complex [41].

### SufC

SufC is encoded along with SufB scaffold in all *suf* operons identified so far, in agreement with biochemical evidences showing that these two proteins interact to form a SufB_2_C_2_ complex (Fig. 3). While it is not clear if this interaction is physiologically relevant in *E. coli*, it reflects the active SufCB complex in organisms that lack SufD and have a minimal *sufBC* operon such as *Methanococcus vulcanius and Blastocystis*. SufC is a monomer in solution (Fig. 3) and is endowed with an ATPase activity [12, 42]. It contains all motifs that are characteristic of the ABC ATPases, like the Walker sites A and B as well as ABC signature [43–45]. The basal ATPase activity of the SufC alone is quite low but significantly enhanced when SufC is associated with either SufB or SufD (180-fold with SufB and fivefold with SufD) [46]. Some amino acids were identified as potentially important for ATPase activity [Lys40, Lys152, Glu171, Asp173 and H203 (*E. coli* SufC)] based on comparison with ABC ATPases, but there was no in vitro study associated. So far, the ATPase activity of SufC was not shown to be important for Fe–S assembly in vitro. However, deletion of *sufC* or mutation in the ATP binding site abolish in vivo function of the SUF pathway [12, 47, 48]. In particular, the as purified His6–SufBC_2_D–SufC(L40R) in which there is a point mutation in the Walker A site of SufC (and thus no ATPase activity) displays a eightfold reduction of iron content relative to the wild-type His6–SufBC_2_D strongly suggesting that the ATPase activity is necessary for iron acquisition in vivo during Fe–S assembly [35]. If the entire *E. coli suf* operon is expressed, SufC is able to associate with SufB and SufD forming the SufBC_2_D complex (Fig. 3).

### SufD

SufD is a paralog of SufB (17% identity and 37% similarity) and sequence homology suggests that its gene derives from a duplication of an ancestral SufB sequence. This is in agreement with phylogenetic analyses showing that SufB gene appears earliest in the evolutionary time among the *suf* genes. SufD from *E. coli*, has a sequence with no known predicted motifs, and after purification from *E. coli* it contains any cofactor or prosthetic group. Even it is a paralog of SufB, after incubation with an excess of iron and sulfide, SufD does not harbor any Fe–S cluster. SufD is stable as purified and under an homogeneous dimeric form (Fig. 3). SufD is proposed to play a role in iron acquisition since deletion of *sufD* diminishes the iron content of the SufB_2_C_2_ sub-complex (like the SufC K40R mutation) [35]. Early studies by F. Barras and Expert’ groups demonstrated a link between SufD and iron metabolism [12, 49]. However, so far, there is no in vitro study showing that SufD binds iron either ferrous or ferric, even transiently. SufD can interact with SufC and SufB to form SufBC_2_D complex and in the absence of SufB can form also a SufC_2_D_2_ complex (Fig. 3) [50].

### SufS

SufS is a PLP-dependent dimeric cysteine desulfurase (Fig. 3) that mobilizes sulfur from l-cysteine substrate, resulting in an enzyme-bound persulfide intermediate at Cys-364 (*E. coli* numeration) in the active site. It belongs the group II desulfurase enzyme family and have low basal activity with regard to group I of cysteine desulfurase family such as IscS. Several structural features distinguish group II enzymes from group I explaining their differences in activity. In particular, a key structural difference between SufS and IscS is that the extended lobe of SufS containing the active site loop has an 11-residue deletion. The shortening of this region in SufS structurally restricts the flexibility of the SufS Cys364-anchoring extended lobe. In contrast, the corresponding loop of IscS is longer and disordered in most structures of IscS due to its flexibility [51]. Therefore, group II cysteine desulfurases require a specific sulfur shuttle protein for full activity. Furthermore, SufS binds tightly to SufE (*K*_D_: 0.36 µM) and the resulting 1:1 complex displays a much larger cysteine desulfurase activity [52, 53]. Molecular investigations demonstrated that sulfur enters at SufS, in the form of persulfide on Cys364, and that thanks to a transpersulfuration reaction sulfur is transferred to the invariant SufE Cys51 residue [54]. The sulfur transfer from SufS to SufE proceeds via a ping-pong mechanism that may be important for limiting sulfur transfer under oxidative conditions [55, 56].

### SufE

SufE protein exists under a monomeric form in solution (Fig. 3). As mentioned above, SufE protein interacts with the SufS dimer in a 1:1 stoichiometry forming in solution a SufS_4_E_4_ complex (J. Pérard, unpublished results) (Fig. 3). When SufE interacts with SufS, the cysteine desulfurase activity is increased by an order of magnitude [52, 54]. The slowest step in the desulfurase activity corresponds to the nucleophilic attack of the Cys364 thiolate on the substrate cysteine-PLP ketimine adduct. The invariant Cys51 of SufE acts as a co-substrate for SufS and accepts the sulfur from Cys364 of SufS, thereby enhancing the catalytic rate [52–55]. Recent investigations show that interaction of SufE–SufS elicits changes in structural dynamics of SufS within its active site facilitating the desulfuration reaction and also that a conformational change of SufE accompanies the interaction with SufS [57]. Thus, coupled conformational changes likely accompanies the SufS–SufE interaction explaining the enhancement of the cysteine desulfurase activity. This is described in more details in the structural section below. Cysteine desulfurase activity of SufSE complex is further enhanced by both SufB_2_C_2_ and SufBC_2_D complexes [33, 53] and recently, residues Cys254, Gln285 and Trp287 of SufB were identified to be critical for the enhancement of the cysteine desulfurase activity of SufSE by SufBC_2_D [41].

### SufU

SufU is present in many bacteria, in particular members of the phylum *Firmicutes* (*Bacillus subtilis*, *Enterococcus faecalis*), and in some *Mycobacteria* (*M. tuberculosis*) (Fig. 2). In *B. subtilis*, it is essential for survival [58, 59]. The SUF pathway of the organisms that contain SufU has SufB, SufC, SufD and SufS but lacks the mandatory sulfur acceptor SufE. Strikingly, genomic analysis showed that SufU and SufE tend not to co-occur (i.e., nearly all species containing *sufU* lack a copy of the *sufE* gene, and vice versa). *B. subtilis* SufU diverges structurally from the SufE-like proteins in that it has two additional cysteine residues that are poised near the sulfur acceptor site (Cys41 in *B. subtilis* SufU). D43A mutation of SufU results in purification of small amounts of Fe–S cluster, proposed to be bound by the three cysteines [59]. The ability of SufU(D43A) to bind small amounts of Fe–S cluster led to propose SufU as an Fe–S scaffold protein for the SUF system in *Firmicutes* [59, 60]. In agreement with this idea, recombinant purified wild-type SufU that is devoid of Fe–S clusters, binds upon in vitro reconstitution a 4Fe–4S cluster under sub-stoichiometric amounts. The cluster can be transferred to the isopropylmalate isomerase Leu1, forming catalytically active Fe–S-containing Leu1 [59]. SufU interacts with SufS (Fig. 3) and activate sulfur transfer by enhancing SufS activity about 40-fold in vitro [59, 61]. Therefore, it was proposed that SufU functions as an Fe–S cluster scaffold protein tightly cooperating with the SufS cysteine desulfurase. This assignment of SufU as a scaffold was consistent with the extensive homology between SufU and the IscU. However, several observations suggest these two proteins have different roles. (1) Sequence alignments reveal small but important differences between IscU and SufU. SufU proteins contain an insertion of 18–21 residues between the second and third cysteine residue, and SufU has also replaced a key histidine residue (H105 of IscU) used for cluster binding. (2) IscU does not enhance the activity of its cognate desulfurase IscS to the same level as SufU does for SufS [52, 58, 61, 62]. (3) The three cysteine residues of *B. subtilis* SufU (Cys41, Cys66, Cys128) together with the Asp43 constitute a binding site for Zn^2+^ [63], that is tightly bound to SufU (*K*_a_ of 10^17^ M^−1^) [16]. Substitution of these amino acids disrupts zinc binding. The enhancement of SufS activity by SufU requires Zn^2+^ to be bound to SufU. Individual Ala-substitutions of Cys41, Cys66, Cys128 and Asp43 eliminate sulfurtransferase activity [16]. It is impossible to reconstitute an Fe–S cluster on a zinc-bound SufU that was shown to stabilize the protein [16]. Based on all these results and considering that there is no need to get two distinct scaffolds (SufU and SufB) on a same SUF pathway, the reasonable current model of SufU function is that it acts as a sulfur transfer partner for SufS but is not a *bona fide* scaffold protein [16]. The precise role of zinc as a structural and/or catalytic element during sulfur transfer reaction remains to be uncovered.

### SufA

SufA is a member of the A-type carrier (ATC) family of Fe–S cluster carrier proteins including IscA and ErpA [64]. SufA is a dimer in solution (Fig. 3) and it shares with IscA the ability to bind 2Fe–2S and 4Fe–4S clusters after chemical reconstitution [65, 66]. When purified anaerobically after co-expression in vivo with its cognate partner proteins from the *suf* operon (SufBCDSE) it contains a 2Fe–2S cluster [67]. Like most of ATC proteins, SufA contains three strictly conserved cysteine residues (C_50_XC_114_XC_116_ for *E. coli* SufA) which are proposed for a long time to act as ligands of the Fe–S cluster based on mutagenesis studies on eukaryotic homologues [68]. However, structural data strongly suggest another coordination mode (see below) [69]. SufA can transfer its cluster to downstream apo-proteins such as biotin synthase, aconitase (4Fe–4S enzymes) and Fdx (2Fe–2S protein) [39, 67]. Cluster transfer from pre-assembled 2Fe–2S SufA to Fdx is more efficient than cluster transfer from 4Fe–4S SufB_2_C_2_ and SufBC_2_D to Fdx. The difference in transfer efficiency between SufA and complexes may be due to the fact that 2Fe–2S cluster of SufA can be directly transferred to Fdx while 4Fe–4S of complexes has to undergo first a conversion step (4Fe–4S to 2Fe–2S) prior to transfer to Fdx [40]. It is also possible that the structure of SufA may promote more rapid release of the 2Fe–2S cluster as compared to complexes. SufA cannot transfer its cluster to SufBC_2_D but on the other hand can receive cluster from SufBC_2_D [39]. Even though SufBC_2_D can transfer Fe–S cluster to Aconitase (4Fe–4S) without requirement of SufA [38], recent studies demonstrated that the cluster transfer to aconitase from SufBC_2_D or SufB_2_C_2_ proceed through a Fe–S SufA intermediate if apo-SufA is present during the Fe–S transfer [40]. This suggests that SufA is important for maturation of 4Fe–4S proteins and that SufA likely provides specific mechanistic advantages for cluster transfer to 4Fe–4S targets proteins under physiological conditions as suggested from genetic data [70]. In conclusion, all studies on SufA are in agreement with the notion that SufA is a Fe–S carrier rather than a Fe–S scaffold protein dedicated to maturation of 4Fe–4S proteins.

## Structural and biophysical analyses of Suf proteins

### SufS

**Fig. 4:**
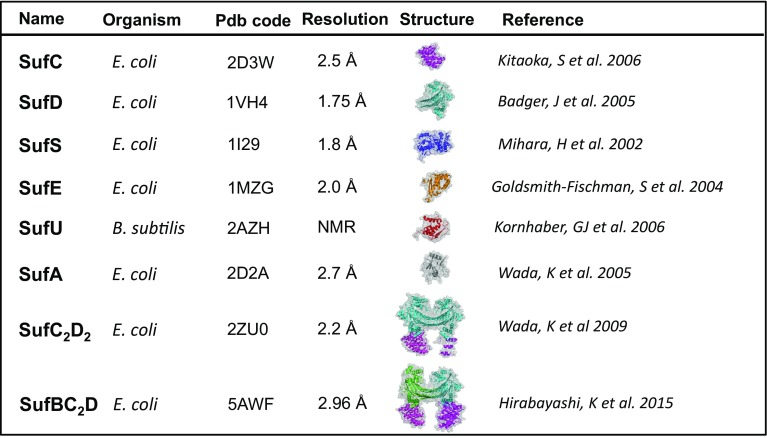
Overview of Suf protein structures

There are five crystal structures of SufS protein (PDB numbers: 5J8Q; 4W91; 1T3I; 5DB5; 1I29) whose three published (Fig. 4) [71–73]. The first crystal structure was obtained in 2002 with SufS from *E. coli* (initially named CsdB) [73]. The Cys364 residue, which is essential for the activity of SufS toward l-cysteine is clearly visible on a loop of the extended lobe (Thr362–Arg375) in all enzyme forms studied, in contrast to the corresponding disordered loop (Ser321–Arg332) of the *T. maritima* NifS-like protein, which is closely related to IscS. The extended lobe of SufS has an 11-residue deletion compared with that of IscS leading to a restricted flexibility of the Cys364-anchoring extended lobe in SufS. Structure of SufS from *Synechocystis* sp. is very similar to that of *E. coli* SufS [71]. It shows that the loop on which the catalytic Cys372 resides is well-ordered and also shorter by 11 residues in comparison to IscS from *T. maritima*. Sequence comparisons establish that all SufS proteins have loops of similar length. The catalytically essential cysteine of SufS is located in a deep cleft, 5 Å away from PLP, in a region of the polypeptide chain with limited flexibility. This might explain why the activity is so weak and why the limiting step of the reaction is the formation of the persulfide at the catalytic cysteine. Very recently, high-resolution crystal structure of the *B*. *subtilis* (Bs) homodimer in its product-bound state (i.e., in complex with pyridoxal-5-phosphate, alanine, Cys361-persulfide) was obtained [72]. Like for other SufS proteins, BsSufS monomer forms a tightly intertwined homodimer with another monomer across the crystallographic symmetry axis. In addition, the interface and architecture of the BsSufS homodimer closely resemble those of *E. coli* SufS.

### SufE/SufU

There are three crystal structures of SufE protein (PDB numbers: 1NI7; 1MZG; 1WL0) [74, 75] under monomeric form (Fig. 4). *Escherichia coli* SufE displays 35% identity with *E. coli* CsdE (YgdK) (PDB id 1NI7). CsdE is a sulfur acceptor protein from CsdA cysteine desulfurase and together they form a complex like SufSE [76]. The structures of *E. coli* SufE (RX) and CsdE (NMR) are strikingly similar, but in spite of their strong structural conservation, there are differences in the protein dynamics in the vicinity of the sulfur-acceptor site in these two proteins, that may be responsible for a differential binding specificity for the desulfurase or for downstream sulfur-acceptor proteins. *E. coli* SufE structure shows that the active cysteine Cys51, forming persulfide, occurs at the tip of a loop, where its side-chain is buried from solvent exposure in a hydrophobic cavity [74]. This orientation of SufE active site cysteine loop might be an advantage since it may protect the protein from oxidation. However, SufE Cys51 must come into close proximity to active Cys364 of SufS for transpersulfuration reaction, and therefore, SufE protein must undergo a conformational change allowing a flexibility of its loop require for sulfur transfer mechanism with SufS. Examination of the structure of the resting SufE shows a variety of interactions that hold the active site loop folded down into the interior of SufE and reveals that the Asp74 residue would play a key role for maintaining such a structure. Amide hydrogen/deuterium exchange mass spectrometry (HDX-MS) analysis of the SufE D74R mutant revealed an increase in solvent accessibility and dynamics in the loop containing the active site Cys51 used to accept persulfide from SufS [77]. In addition, SufE D74R mutant is a better sulfur acceptor for SufS than wt SufE. Therefore, D74R substitution induces a conformational change in SufE, making the Cys51 active site loop more dynamic for sulfur transfer mechanism. Since Asp74 is located in the peptide 66–83 of SufE that interacts with SufS [57], it is proposed that D74R mutation mimics SufE–SufS interaction leading conformational changes that are propagated to the Cys51 loop allowing transpersulfuration reaction between SufS and SufE. We will see below that indeed, interaction of SufS with SufE leads to a similar phenomenon [57].

Concerning SufU, there is only one structure from *B. subtilis* (PDB code: 2AZH) (Fig. 4). The structure shows the presence of the zinc atom bound to SufU that displays a tetra-coordination by the four conserved residues, Cys41, Cys66, Cys128, and Asp43 [63].

### SufSE complex

**Fig. 5:**
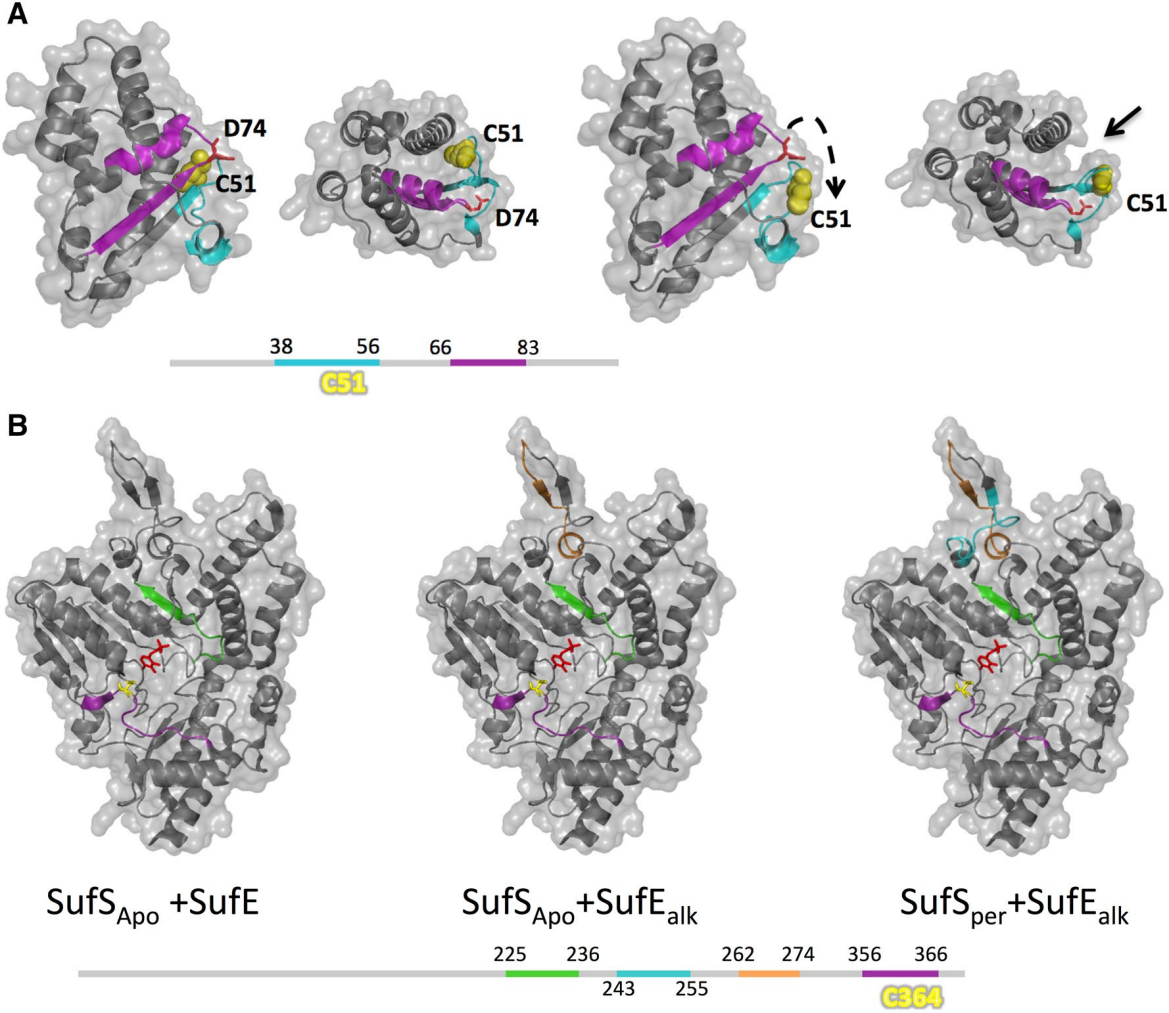
Interactions studies of SufS–SufE by HDX-MS. **a** Effect of SufS on SufE protein by HDX trapping assays. The model represents SufE protein before (left panels, two orientations) and after (right panels, two orientations) interaction with SufS. At the bottom of **a** is represented linear SufE sequence with important peptides whose accessibility to deuterium is modified by SufS interaction. The interaction between SufE and SufS implicated deuterium protection of peptide 38–56 (cyan) containing C51 and peptide 66–83 (magenta) of SufE. The C51 flipping process in the presence of SufS (represented by the black hatched arrow) leads to C51 solvent accessibility and the formation of a groove (black arrow) (manual representation by pymol). **b** Effect of SufE on SufS protein by HDX trapping assays. The model represents SufS protein. At the bottom of **b** is represented linear SufS sequence with important peptides whose accessibility to deuterium is modified by SufE interaction. The persulfide C364–SSH is indicated (in yellow) closed to the PLP cofactor labeled in red. Interaction between SufS_Apo_ and SufE implicated deuterium protection of peptide 225–236 (green) and 356–366 (magenta). Interaction between SufS_Apo_ and SufE_alk_ implicated deuterium protection of peptides 225–236 (green), 262–274 (orange) and 356–366 (magenta). Interaction between SufS_per_ and SufE_alk_ implicated deuterium protection of peptides 225–236 (green), 262–274 (orange) and 356–366 (magenta) and increase accessibility of peptide 243–255 (cyan)

There is no SufS–SufE three-dimensional structure making it difficult to understand the SufS–SufE sulfur transfer reaction at the molecular level and the origin of the stimulating effects of SufE on the SufS cysteine desulfurase activity. However, recently some HDX-MS and deuterium trapping experiments have been carried out on *E. coli* SufE and SufS proteins as a reporter of protein–protein interaction zones and conformational changes, providing mechanistic insights into the sulfur transfer and enhancement of the cysteine desulfurase activity [57]. These studies indicate that SufE interacts with SufS via two peptides: peptide 38–56 (a surface loop containing Cys51) and peptide 66–83 (that forms one side of a structural groove into which Cys51 thiolate is oriented) (Fig. 5a). Interaction of SufE–SufS induces some conformational changes on SufE, in particular at the level of the Cys51 loop whose solvent accessibility is increased upon SufS binding [57]. SufE carrying D74R mutation (see above), localized in peptide 66–83, prevents hydrogen bond with peptide 38–56, destabilizing interaction between the active site loop and the interior groove. This induces a SufE conformational change by making the Cys51 active site loop more dynamic. In addition, it was shown that this mutation promotes higher interaction of SufE with SufS [77]. Therefore, this mutation enhances the ability of SufE to accept sulfur from SufS. All this suggests that SufE active Cys51 becomes accessible for sulfur transfer after activation due to a conformational change induced by SufS through peptide 66–83 of SufE.

SufE interaction to SufS induces localized dynamic perturbations on SufS (Fig. 5b) involving PLP binding site and active site cysteine 364 loop, however, without inducing global conformational changes on SufS. Indeed, interaction between SufS and SufE implicates deuterium protection of peptides 225–236 and 356–366. Interaction between SufS and SufE_alk_ (where the SufE catalytic cysteine was alkylated mimicking a sulfur-accepting conformation) implicates deuterium protection of peptides 225–236, 262–274 and 356–366. Finally, interaction between SufS_per_ (SufS containing persulfide) and SufE_alk_ implicates deuterium protection of peptides 225–236, 262–274 and 356–366 and an increased accessibility of peptide 243–255. All these results suggest that the presence of SufE (1) promotes external aldimine formation between PLP and l-cysteine, and therefore, persulfide formation Cys364 of SufS and (2) diminishes the persulfide stabilization facilitating the nucleophilic attack by SufE Cys-51 on the SufS Cys-364 persulfide for direct sulfur transfer. Finally, these studies demonstrate that once SufS and SufE interact some subtle dynamics exist which are the molecular basis explaining the sulfur transfer between SufS and SufE reaction and the enhanced cysteine desulfurase activity.

Recently, crystallographic structure of the *E. coli* CsdA–CsdE complex was solved [78]. Since all active cysteine-containing regions are well ordered it was possible to compare the structure of the complex to that of structures of free CsdA and CsdE proteins [75, 78]. In comparison with SufS–SufE complex some similarities can be drawn. Like for the SufE within the SufS–SufE complex, in the CsdA–CsdE structure, the CsdE Cys-61 loop region moves and becomes exposed. It undergoes an 11 Å shift upon interaction with CsdA becoming oriented toward Cys-358 of CsdA for sulfur transfer. The distance between the two active cysteines of CsdA and CsdE is estimated to be 6 Å. Given that the transition state of the transpersulfuration reaction contains three sulfur atoms and that the disulfide bond length is 2.1–2.3 Å, these two captured cysteines of CsdA and CsdE in the structure are likely of the intermediate stage. Despite the change in CsdE conformation, there are no noticeable structural changes to the CsdA cysteine desulfurase backbone in the CsdA–CsdE complex like for SufS in the presence of SufE.

HDX-MS experiments were also initiated on *B*. *subtilis* SufS and SufU. Binding of BsSufU to BsSufS induces conformational changes in both proteins [72]. These experiments demonstrate that SufU induces an opening of the active site pocket of SufS allowing the Cys361 loop of SufS to move freely [72].

### SufC

**Fig. 6:**
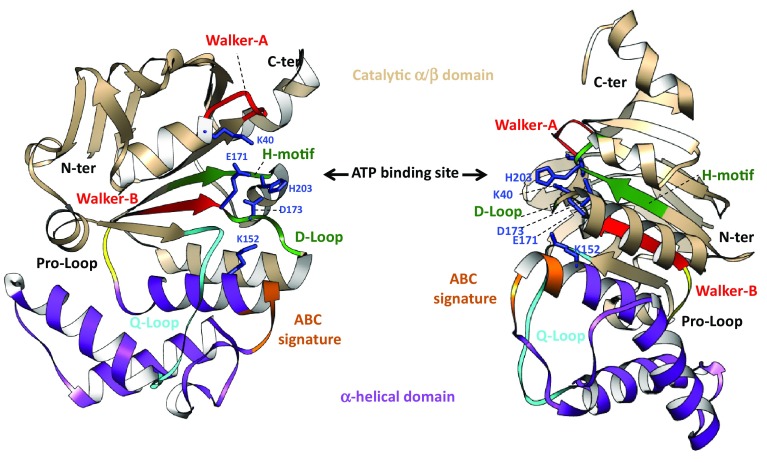
Structure detail of SufC protein (pdb code 2D3W). Critical domains and important regions are illustrated into the structure. The ATP binding site are indicated by black arrow and critical amino acid residues were indicated in blue. Picture is obtained by Chimera (1.10.2)

There are two crystal structures of monomeric SufC protein from *Thermus thermophilus* HD8 and *E*. *coli* (PDB numbers: 2D2F; 2D3W) (Fig. 4) [79, 80]. The SufC subunit has two domains, as observed in the members of the ABC ATPase family: a catalytic α/β domain that contains the nucleotide-binding Walker A and Walker B motifs, and a helical domain specific to ABC ATPases containing an ABC signature motif. The two domains are connected by a Q-loop that contains a strictly conserved glutamine residue (Fig. 6). The overall architecture of the SufC structure is similar to other ABC ATPases structures, but there are several specific motifs in SufC. Indeed, the structure of SufC reveals an atypical nucleotide binding conformation at the end of the Walker B motif. Three residues following the end of the Walker B motif form a novel 3_10_ helix (type of secondary structure) which is not observed in other ABC ATPases. Due to this novel 3_10_ helix, the conserved glutamate residue (Glu169 in *T. thermophiles*, Glu171 in *E. coli*) involved in ATP hydrolysis is flipped out. Although this unusual conformation is unfavorable for ATP hydrolysis, it is stabilized by several interactions around the novel 3_10_ helix. Glu and Asp residues (Glu169 and Asp171 in *T. thermophiles*, Glu171 and Asp173 in *E. coli*) form salt-bridges with a Lysine (Lys150 in *T. thermophiles*, Lys152 in *E. coli*); and there are several water molecules that form a strong hydrogen bond network. This makes the novel 3_10_ helix of SufC a rigid conserved motif [80].

In addition, compared to other ABC ATPase structures, a significant displacement occurs at a linker region between the ABC α/β domain and the α-helical domain. The linker conformation is stabilized by a hydrophobic interaction between conserved residues around the Q loop. Finally, the surface of SufC has a cleft different from those observed in other ABC ATPase structures. These results suggest that SufC interacts with its partners, SufB and SufD, in a manner different from that of ABC transporters.

### SufD

There is one crystal structure of SufD protein (PDB number: 1VH4) (Fig. 4) [81]. SufD displays a novel structure and forms a crystallographic dimer. It shares 20% identity with SufB, and therefore, likely share a similar fold. This novel structure of SufD is a flattened right-handed beta-helix of nine turns with two strands per turn; the N- and C-termini form helical subdomains. Homodimerization of SufD doubles the length of the beta-helix (to 80 Å) and two highly conserved residues, Pro347 and His360, interact at the dimer interface. There are several highly conserved residues in the C-terminal subdomain (Tyr374, Arg378, Gly379, Ala385, Phe393), whose role is unknown. All these residues mentioned are conserved in SufB, supporting the hypothesis that it is able to homodimerize in a similar manner to SufD and that in vivo SufB and SufD may form a functional heterodimer analogous to the SufD homodimer. This heterodimer SufD–SufB exists within the SufBC_2_D complex [37] with a structure of SufD almost identical to the reported SufD heterodimer. SufD is also able to interact with SufC to form a SufC_2_D_2_ sub-complex [50].

### SufC_2_D_2_ complex

As mentioned before SufC and SufD interact forming a SufC_2_D_2_ complex [46, 50] whose stoichiometry was determined by mass spectrometry and light scattering experiments. Electron microscopy and X-ray crystallography structures of the SufC_2_D_2_ complex from *E. coli* were determined (Fig. 4) [50]. Knowing that the minimalist *suf* operon contains only sufB and sufC genes, this structure has probably no physiological significance but it likely mimics the quaternary structure of a SufB_2_C_2_ or a SufBC_2_D complex considering the sequence similarity between SufB and SufD proteins. Therefore, the SufC_2_D_2_ complex structure constitutes an informative structure for the understanding of Fe–S biogenesis.

In the structure of SufC_2_D_2_, though each SufC subunit is bound to each subunit of SufD homodimer, one SufC subunit was mostly disordered. Since the SufC_2_D_2_ complex exhibits an apparent twofold symmetry, the invisible segments of the SufC subunit were modeled. The model structure of the SufC_2_D_2_ complex is in agreement with the 3D-reconstitution image of the complex derived from negative-stain electron microscopy confirming the quaternary structure of the SufC_2_D_2_ complex.

The structure of the SufD homodimer in the SufC_2_D_2_ complex is almost identical to that reported for SufD homodimer crystallized alone. Its C-terminal part interacts with SufC via extensive hydrophobic interactions as well as hydrogen bonds and one salt-bridge. Interestingly, the SufD residues involved in the hydrophobic interactions are conserved not only in SufD orthologues but also in SufB sequences. The helices in the C-terminal helical domain of SufD interact with the β6 strand, the α2 and α3 helices and the Q-loop of SufC, which are located between the α/β and helical domains of SufC. SufC and SufD interact through extensive hydrophobic interactions as well as by eight hydrogen bonds and one salt-bridge. Although the overall structure of SufC in the SufC_2_D_2_ complex is similar to that of the monomeric form previously reported, several significant structural changes occur in the ATP-binding segments upon complex formation. Importantly, the unique salt bridge observed in the monomeric *E. coli* SufC between Glu171 in the Walker B motif and Lys152 is cleaved, allowing the rotation of the Glu171 side-chain toward the ATP-binding pocket. His203, another key residue for the activity of ABC ATPases, is shifted ± 5 Å toward Glu171. These structural changes remodel the catalytic pocket of SufC to be suitable for ATP binding and hydrolysis and result in a SufC local structure that more closely resembles that of active ABC-ATPases. Thus, as a monomer SufC is in a latent form associated with a weak ATPase activity, whereas in complex with SufD it represents a competent active form. These observations are consistent with the kinetic experiments reporting that ATPase activity of SufC is enhanced by SufD [46, 82]. Finally, in the SufC_2_D_2_ structure the two SufC subunits are spatially separated. Cross-linking experiments performed in solution indicate that the two SufC subunits can associate with each other in the presence of Mg^2+^ and ATP [80]. Therefore, a transient dimer formation of SufC can occur during ATP binding and hydrolysis and likely elicits a significant conformational change of the entire SufC_2_D_2_ complex.

As a conclusion from the SufC_2_D_2_ structure, mainly information got from SufC are of significant importance for Fe–S biogenesis process. The SufC sequence possesses several motifs: those that contribute to ATP binding and hydrolysis (Walker A, Walker B, and ABC signature), one for dimerization (D-loop), and one for interaction with partner proteins (Q-loop). These properties are encountered also in the SufBC_2_D structure.

### SufBC_2_D complex

As mentioned before SufB, SufC, and SufD interact with each other generating a SufBC_2_D complex whose stoichiometry was determined by mass spectrometry [38]. Formation of the SufBC_2_D complex results from the controlled expression from the intact *sufABCDSE* operon (and not from incubation between SufB, SufC and SufD purified proteins). Under these conditions, no SufC_2_D_2_ complex is detected and small amount of SufB_2_C_2_ complex is observed but still contaminated with SufD (stoichiometry 0.5) [35]. This likely indicates that the physiological and active complex for Fe–S biogenesis in *E. coli* is the ternary SufBC_2_D complex. This is in agreement with in vivo and in vitro studies which show that SufBC_2_D complex plays a central role in Fe–S assembly and is the platform for Fe–S cluster assembly [12, 38, 39, 53]. For a long time, getting structural information of the SufBC_2_D complex was impossible, and therefore, considered as a real challenge. Recently, the structure of the *E. coli* SufBC_2_D complex was solved at 2.95 Å resolution (Fig. 4) [37]. It consists of one SufB subunit, two SufC subunits, and one SufD subunit with a stoichiometry of 1:2:1, consistent with previous biochemical experiments [38]. This structure does not reveal any cofactors such as Fe–S cluster and/or FADH_2_ that bind the SufBC_2_D complex after anaerobic purification [38]. Negative-stain electron microscopy and small angle X-ray scattering (SAXS) data from the as-isolated SufBC_2_D complex in solution are in agreement with the crystal structure [37]. As expected from the SufC_2_D_2_ structure, the SufBC_2_D complex shares a common architecture with SufC_2_D_2_ where one SufD subunit is replaced by the SufB subunit and SufB interacts with both SufD and SufC. Thus, each of the SufC subunits is bound to a subunit of the SufB–SufD heterodimer (termed SufC_SufB_ and SufC_SufD_). SufC_SufB_ and SufC_SufD_ have almost identical structures. On the whole, structure of SufBC_2_D is very similar to that of SufC_2_D_2_ [50] as follow: (1) the two SufC subunits are bound (one with SufB and one with SufD) but spatially separated (more than 40 Å) with their ATP-binding motifs facing one another. Each SufC subunit can transiently associate with each other in the complex in the presence of Mg^2+^ and ATP as shown by disulfide cross-linking experiment; (2) the overall structure of SufC subunits in the SufBC_2_D complex is similar to that of monomeric SufC (51) with significant structural changes around the ATP-binding pocket: (a) the salt bridge observed in the monomeric SufC between Glu171 and Lys152 is cleaved in the complex, leading to the rotation of the Glu171 side chain toward the ATP-binding pocket; (b) His203 is shifted about 4 Å toward Glu171 in the complex. These structural changes rearrange the catalytic pocket of SufC to be suitable for ATP binding and hydrolysis. These findings are consistent with kinetic experiments showing that the SufC ATPase activity is enhanced by the presence of SufB and SufD [46, 82]; (3) structure of the SufD subunit is almost identical to that of one subunit of the SufD homodimer [81].

Concerning specific features encountered in the SufBC_2_D complex. The structures of SufB and SufD are similar and share a common domain organization: an N-terminal helical domain, a core domain which consists of a right-handed parallel β-helix, and a C-terminal helical domain to which SufC interacts. Important structural change of the SufBC_2_D complex occur, initiated by SufC dimerization in the presence of Mg^2+^ and ATP. Thanks to a fluorescent experiment, Cys405 of SufB, a strictly conserved amino acid buried at the heterodimer interface between the SufB and SufD heterodimer, was shown to become exposed during ATP hydrolysis. His360 of SufD, localized close to Cys405 of SufB, likely undergoes similar exposure upon conformation change. Finally, two Hg^2+^ ions are present in the structure at the interface of the SufB–SufD heterodimer. One bound to Cys405 in SufB, and the other bound to Cys358 in SufD, which is located adjacent to His360 of SufD. These ions can bind the authentic Fe–S binding site, and therefore, these three residues Cys405 in SufB, H360 and Cys358 in SufD were proposed as good candidate for Fe–S cluster ligation [37]. We will see that in vivo experiments excluded Cys358 of SufD (see below).

As a conclusion, the main insight brought by the SufBC_2_D structure in comparison to SufC_2_D_2_ structure is that SufC forms a transient head-to-tail dimer within the complex during the catalytic step of ATP binding and hydrolysis and that SufC dimerization drives huge structural changes of the SufB–SufD heterodimer, leading to the exposure of Cys405 of SufB inside the heterodimer interface (and likely H360 of SufD). At this stage, the Fe–S assembly story would be the following. In the resting state, the SufC in the complex is ready for ATP binding, and the nascent cluster-assembly site at the SufB and SufD interface is buried inside the complex. Upon ATP binding, SufC forms the head-to-tail dimer and its dynamic motion is transmitted to the SufB–SufD heterodimer where the invariant residue Cys405 in SufB and likely the His360 in SufD, become exposed to the surface to construct the nascent Fe–S cluster.

### SufA

There is one crystal structure of *E. coli* SufA protein (PDB number: 2D2A) (Fig. 4) [69]. The structure corresponds to an apo-form of the protein, without Fe–S cluster. SufA shares 48% sequence identity with IscA but SufA exists in crystals as a homodimer, in contrast to the tetrameric organization of apo-IscA [83]. Furthermore, the C-terminal segment containing two essential cysteine residues (Cys–Gly–Cys), which is disordered in the IscA structure, is clearly visible in one molecule (the α1 subunit) of the SufA homodimer. Although this segment is disordered in the other molecule (the α2 subunit), computer modeling suggests that the four cysteine residues of the Cys–Gly–Cys motif (Cys114 and Cys116 in each subunit) are positioned in close proximity (3.1–6.7 Å) at the dimer interface allowing in SufA dimer coordination of an Fe–S cluster. More recently, the crystal structure of a 2Fe–2S cluster-bound form of *Thermosynechococcus elongatus* IscA showed a different coordination mode. Indeed, the structure is formed by an asymmetric, domain-swapped tetramer formed by two α and two β subunits, in which the 2Fe–2S cluster is coordinated by two conformationally distinct α and β subunits, with asymmetric cluster coordination by Cys37, Cys101, Cys103 from α and Cys103 from β [84]. Later, the domain swapping has been attributed to a crystallization artifact [85]. Very recently, a nice work performed by NMR demonstrates that the 2Fe–2S cluster on the human ISCA2 homodimer is coordinated transiently by Cys144 and Cys146 of each monomer and that this form evolves to a more thermodynamically species in which the 2Fe–2S cluster is ligated by Cys79 and Cys144 [86]. It is possible that a similar coordination exist on SufA containing a 2Fe–2S cluster.

## In vivo studies on SufBC_2_D

Since the beginning of Fe–S assembly study, many in vivo experiments were carried out on the *suf* operon. They mainly consisted in studying the in vivo impact (Fe–S enzymes activity, bacterial growth, sensitivity to oxidants and iron chelator…) after inactivation of a single *suf* gene and allowed to demonstrate that *suf* operon is involved under oxidative stress and iron limitation [12, 24, 42, 49, 87]. In the next lines, we will focus on the impact of point mutations in sufB, sufC or sufD genes within the *suf* operon on the Fe–S assembly process in *E. coli*. To detect the effect of mutation in vivo, two strategies were used. One strategy was to perform complementation assays using an *E. coli* mutant strain that can survive without Fe–S clusters [88]. In this *E. coli* strain (UT109) the chromosomal *suf and isc* operons are deleted (Δ*sufABCDSE* Δ*iscUA*-*hscBA*). Deletion of both operons in *E. coli* is lethal in general; but, UT109 harbors the plasmid pUMV22 that carries three genes for the mevalonate (MVA) pathway, which allows UT109 to grow with an absolute dependence on MVA supplementation [88]. Upon introduction of functional sufAB and sufCDSE genes (via plasmids) the cells become able to grow normally even in the absence of MVA. Therefore, this strategy highlights crucial amino acid of the SUF system for Fe–S metabolism in *E. coli*. The second strategy consists to assess Fe–S assembly in vivo on SufBC_2_D using the color of host cells overproducing SufBC_2_D complex that contain mutation on SufB, SufC or SufD proteins. Cells are blackish-green when active SufBC_2_D complex is overproduced and white for an inactive complex, unable to form a Fe–S cluster [37]. Therefore, this strategy highlights crucial amino acid for Fe–S assembly on SufBC_2_D complex. For both strategies, a series of mutations was generated on SufB, SufC and SufD proteins. The in vivo complementation assays reveal critical amino acids on SufC: Lys40, Glu171 and His203 [37] in agreement with previous experiments showing that Lys40 is essential for SufC ATPase activity and Fe–S formation on SufBC_2_D complex [35]. The second strategy confirms these results since mutants in these amino acids have white cells [37]. Altogether, these results on SufC show that residues Lys40, Glu171 and His203 are essential for the assembly of Fe–S cluster on SufBC_2_D (Table 1).

**Table 1:** Critical amino acid of the SufBC_2_D complex for Fe–S assembly and binding and their proposed function

SufB	
Cys254	Sulfur entry
Cys405	Final sulfur acceptor and Fe–S ligand
Asp432	Potential Fe–S ligand
His433	Potential Fe–S ligand
Asp434	Fe–S ligand
Q285	Sulfur production on SufSE and sulfur channeling
W287	Sulfur production on SufSE
K303	Sulfur channeling
SufD	
His360	Iron acquisition, Fe–S ligand
SufC	
Lys40	ATP hydrolysis
Glu171	ATP hydrolysis
His203	ATP hydrolysis

For SufD, the in vivo complementation assay and cells color reveals that His360 is critical for Fe–S metabolism and Fe–S assembly on SufBC_2_D [37, 41] in agreement with previous data that indicated His360 residue essential for SufD function [37, 50] (Table 1). By this technics, no other residue of SufD were identified as important. Even Cys358, that was shown to be involved in the binding site for one Hg^2+^ ions in the SufBC_2_D structure [37] and that is localized at the SufD–SufB interface, does not prove to be necessary in the complementation assay [41]. It is also not involved in Fe–S assembly on SufBC_2_D since cells overproducing SufBC_2_D(C358A) proteins are blackish-green [37].

For SufB, the in vivo complementation assay revealed that Cys254, Cys405, Arg226, Asn228, Gln285, Trp287, Lys303 and Glu434 are critical for growth (Table 1) [41]. Gln285 and Lys303 take part of a putative tunnel ranging through the α-helix core domain of SufB connecting Cys254 and Cys405 in SufB. Interestingly, deletion of the entire CxxCxxxC canonical motif has no effect on the complementation showing that the three cysteines of this motif are dispensable in vivo and thus not the Fe–S ligands. Interestingly also, is the partial complementation of UT109 strain with the double SufB Glu432A/His433A protein that contains mutations at the SufB–SufD interface [41]. The second strategy, as expected, reveals that SufB Cys405 is important for SufB function since complex containing Cys405A mutation has white cells, indicating that this residue is indispensable for cluster assembly. No experiments were performed with mutations on residues Cys254, Arg226, Asn228, Gln285, Trp287, Lys303 and Glu434.

## Proposed model for Fe–S biogenesis by SUF

Based on biochemical, biophysical, structural and in vivo experiments the current model for Fe–S assembly on SufBC_2_D complex is the following (*E. coli* numeration).

**Fig. 7:**
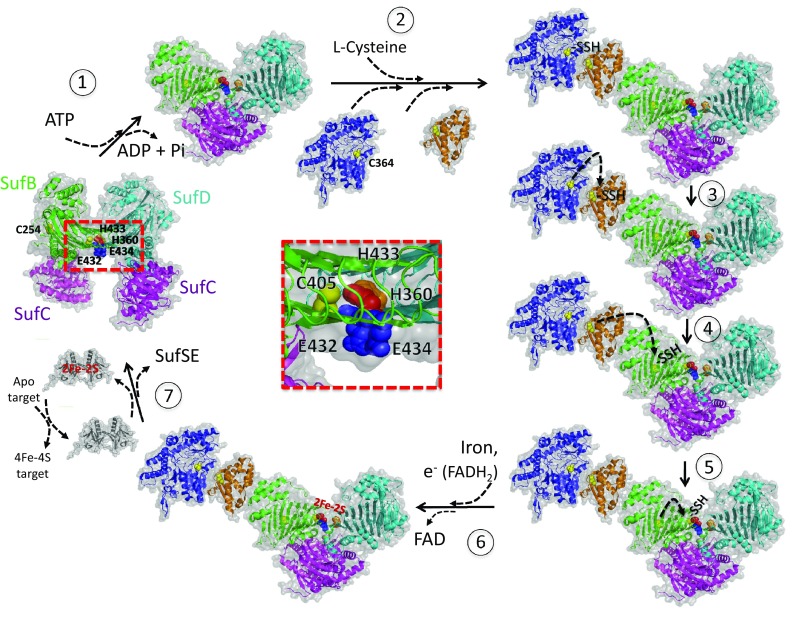
Current proposed mechanism for Fe–S assembly by the SUF system. SufBC_2_D complex is in a relaxation mode. (1) The mechanism is initiated by ATPase activity of SufC. Upon ATP binding, SufC transiently forms a dimer that elicits a significant conformational change of the entire SufBC_2_D complex. The SufB C405 and likely SufD H360 become exposed to the surface. This confirmation of the complex is favorable to recruit SufE–SufS complex. (2) Cysteine desulfurase activity (arrival of l-cysteine) generates a persulfide on SufS (C364) that is transferred to C51 of SufE (3) and then to C204 (4) and C405 (5) of SufB. (6) Arrival of iron and electrons (FADH2) allows building of Fe–S cluster on the complex at the SufB–SufD interface. SufD H360, SufB C405, E434 and H433 or E432 can be involved in Fe–S coordination or Fe–S formation. (7) SufSE release allows transfer of the SufBC_2_D cluster to SufA that can maturate 4Fe–4S target proteins. The original apo-SufBC_2_D complex is regenerated and ready for a new cycle. Critical amino acid are represented with bowls. Amino acids localized at the SufB–SufD interface (red square) are zoomed in the inset in the middle of the figure. SufU, not represented, is suggested to play a SufE-like role

It is likely that the Fe–S assembly is initiated by ATPase activity of SufC. Indeed, in the resting state, the SufC is ready for ATP binding, and the nascent cluster-assembly site at the SufB and SufD interface is buried inside the complex. Upon ATP binding, SufC transiently forms a dimer that elicits a significant conformational change of the entire SufBC_2_D complex. In particular, the invariant residue Cys405 in SufB and likely the His360 in SufD, become exposed to the surface to construct the nascent Fe–S cluster. The building of the Fe–S is possible by arrivals of sulfur and iron ions. SufS catalyzed desulfurization of l-cysteine with the formation of persulfide on its Cys364. Nucleophilic attack of SufE Cys51 thiolate allows transpersulfuration reaction to occur and formation of a persulfide on SufE Cys51. A second transpersulfuration between SufE and SufB generates a persulfide on SufB Cys254 residue that serves as the first sulfur acceptor site on SufB. Then, sulfur migrates from SufB Cys254 to SufB Cys405. SufB Cys405 is > 25 Å away from SufB Cys254. An internal hydrophilic tunnel ranging through the β-helix core domain of SufB just between SufB Cys254 and SufB Cys405 may help during sulfur transfer between these two cysteines. Residues Lys303 and Gln285 might be directly involved (with SufB Glu236, SufB Glu252, SufB His265, SufB Thr283, SufB Thr326 and SufB Lys328). If this putative sulfur tunnel is involved in sulfur transfer from Cys254 to Cys405 (that is an interesting hypothesis) that would be the first time that sulfur transfer reaction occurs without transpersulfuration mechanism, which usually is used for sulfur as a strategy to travel long distances under a non-toxic form. SufB Cys405 is the final sulfur acceptor and a good candidate for one of the Fe–S cluster ligands. SufD His360 is likely another one. SufB Glu434 and SufB His433 or SufB Glu432 may be also involved during Fe–S assembly or as Fe–S ligands (Fig. 7), hypothesis that have to be experimentally tested in a near future.

There are still some remaining questions. How and when iron is delivered to the Fe–S assembly site? is there a specific iron donor protein for the SUF system? Genetic experiments strongly suggest a link between SufD and iron metabolism [12, 35, 49]. However, so far such an hypothesis was not validated in vitro. A flavin is co-purifying under anaerobic conditions with SufBC_2_D complex with a stoichiometry of 1 flavin per complex [38]. Only the reduced form of the flavin (FADH2) binds to the complex, FAD is unable to. We demonstrated in vitro a ferric reductase activity of the flavin (Fe^3+^–Fe^2+^) on small chelates (ferric citrate) and proposed that it can be involved during Fe–S assembly in the reduction of ferric iron [38]. Recently, it was proposed that the flavin can provide electrons for persulfide cleavage (S^0^ to S^2−^) even though this was not demonstrated experimentally [41]. Thus, the actual hypothesis is that FADH_2_ serves to reduce iron. Considering that SUF system is involved under oxidative stress and iron limitation another possibility would be that the reduced flavin serves as a sensor of oxidative conditions (hypothesis never considered so far). The binding site of the FADH_2_ is still unknown despite several experiments using mutants in SufB [41]; and therefore, the assignment of the FADH_2_ binding site requires further studies. Another next challenge in the future in the Fe–S assembly field involving SUF system is to get structural information of an integrated system containing SufSE–SufBC_2_D proteins. The SufSE complex interacts with SufBC_2_D complex to provide sulfur atoms for Fe–S cluster assembly. Sulfur atoms enter SufBC_2_D complex via SufB protein. Some ITC experiments demonstrated a flip–flop mechanism of allosteric regulation where binding of one SufE to one active site of SufS dimer diminishes further SufE binding to the second active site [57]. One can wonder under which oligomerization state SufS–SufE complex interacts with SufBC_2_D complex for sulfur transfer: SufS_2_E, SufS_2_E_2_, SufSE? It is reasonable to hypothesize that a stoichiometric SufSE complex is relevant for interaction with SufBC_2_D since only one sulfur entry to SufB is require (Fig. 7). Another important question is related to the event that drives the interaction between SufSE and SufBC_2_D complexes? As a consequence, it is urgent to stabilize and get structural information on the huge SufSEBC_2_D complex. This would allow to trap Fe–S intermediate, and therefore, identify Fe–S coordination sites and fully understand the Fe–S assembly mechanism.

## References

[1] Beinert H (2000). J Biol Inorg Chem..

[2] Fontecave M (2006). Nat Chem Biol.

[3] Lill R (2009). Nature.

[4] Py B, Moreau PL, Barras F (2011). Curr Opin Microbiol.

[5] Jacobson MR, Cash VL, Weiss MC, Laird NF, Newton WE, Dean DR (1989). Mol Gen Genet.

[6] Frazzon J, Dean DR (2003). Curr Opin Chem Biol.

[7] Zheng L, Cash VL, Flint DH, Dean DR (1998). J Biol Chem.

[8] Lill R, Dutkiewicz R, Elsasser HP, Hausmann A, Netz DJ, Pierik AJ, Stehling O, Urzica E, Muhlenhoff U (2006). Biochim Biophys Acta.

[9] Takahashi Y, Tokumoto U (2002). J Biol Chem.

[10] Balk J, Pilon M (2011). Trends Plant Sci.

[11] Lill R, Hoffmann B, Molik S, Pierik AJ, Rietzschel N, Stehling O, Uzarska MA, Webert H, Wilbrecht C, Muhlenhoff U (2012). Biochim Biophys Acta.

[12] Nachin L, Loiseau L, Expert D, Barras F (2003). Embo J.

[13] Olson JW, Agar JN, Johnson MK, Maier RJ (2000). Biochemistry.

[14] Stehling O, Wilbrecht C, Lill R (2014). Biochimie.

[15] Sheftel A, Stehling O, Lill R (2010). Trends Endocrinol Metab.

[16] Selbach BP, Chung AH, Scott AD, George SJ, Cramer SP, Dos Santos PC (2014). Biochemistry.

[17] Mashruwala AA, Pang YY, Rosario-Cruz Z, Chahal HK, Benson MA, Mike LA, Skaar EP, Torres VJ, Nauseef WM, Boyd JM (2015). Mol Microbiol.

[18] Huet G, Daffe M, Saves I (2005). J Bacteriol.

[19] Charan M, Singh N, Kumar B, Srivastava K, Siddiqi MI, Habib S (2014). Antimicrob Agents Chemother.

[20] Di Perri G, Bonora S (2004). J Antimicrob Chemother.

[21] Choby JE, Mike LA, Mashruwala AA, Dutter BF, Dunman PM, Sulikowski GA, Boyd JM, Skaar EP (2016). Cell Chem Biol.

[22] Roche B, Aussel L, Ezraty B, Mandin P, Py B, Barras F (2013). Biochim Biophys Acta.

[23] Johnson DC, Dean DR, Smith AD, Johnson MK (2005). Annu Rev Biochem.

[24] Outten FW, Djaman O, Storz G (2004). Mol Microbiol.

[25] Lee KC, Yeo WS, Roe JH (2008). J Bacteriol.

[26] Nesbit AD, Giel JL, Rose JC, Kiley PJ (2009). J Mol Biol.

[27] Desnoyers G, Morissette A, Prevost K, Masse E (2009). EMBO J.

[28] Lee JH, Yeo WS, Roe JH (2004). Mol Microbiol.

[29] Boyd ES, Thomas KM, Dai Y, Boyd JM, Outten FW (2014). Biochemistry.

[30] Outten FW (2015). Biochim Biophys Acta.

[31] Mettert EL, Kiley PJ (2015). Biochim Biophys Acta.

[32] Fontecave M, Choudens SO, Py B, Barras F (2005). J Biol Inorg Chem.

[33] Layer G, Gaddam SA, Ayala-Castro CN, de Choudens SO, Lascoux D, Fontecave M, Outten FW (2007). J Biol Chem.

[34] Tsaousis AD, de Choudens SO, Gentekaki E, Long S, Gaston D, Stechmann A, Vinella D, Py B, Fontecave M, Barras F (2012). Proc Natl Acad Sci USA.

[35] Saini A, Mapolelo DT, Chahal HK, Johnson MK, Outten FW (2010). Biochemistry.

[36] Blanc B, Clemancey M, Latour JM, Fontecave M, de Choudens SO (2014). Biochemistry.

[37] Hirabayashi K, Yuda E, Tanaka N, Katayama S, Iwasaki K, Matsumoto T, Kurisu G, Outten FW, Fukuyama K, Takahashi Y (2015). J Biol Chem.

[38] Wollers S, Layer G, Garcia-Serres R, Signor L, Clemancey M, Latour JM, Fontecave M, de Choudens SO (2010). J Biol Chem.

[39] Chahal HK, Dai Y, Saini A, Ayala-Castro C, Outten FW (2009). Biochemistry.

[40] Chahal HK, Outten FW (2012). J Inorg Biochem.

[41] Yuda E, Tanaka N, Fujishiro T, Yokoyama N, Hirabayashi K, Fukuyama K, Wada K, Takahashi Y (2017). Sci Rep.

[42] Nachin L, El Hassouni M, Loiseau L, Expert D, Barras F (2001). Mol Microbiol.

[43] Wilken S, Schmees G, Schneider E (1996). Mol Microbiol.

[44] Zaitseva J, Jenewein S, Jumpertz T, Holland IB, Schmitt L (2005). EMBO J.

[45] Schmitt L, Tampe R (2002). Curr Opin Struct Biol.

[46] Petrovic A, Davis CT, Rangachari K, Clough B, Wilson RJ, Eccleston JF (2008). Protein Sci.

[47] Xu XM, Moller SG (2004). Proc Natl Acad Sci USA.

[48] Gisselberg JE, Dellibovi-Ragheb TA, Matthews KA, Bosch G, Prigge ST (2013). PLoS Pathog.

[49] Expert D, Boughammoura A, Franza T (2008). J Biol Chem.

[50] Wada K, Sumi N, Nagai R, Iwasaki K, Sato T, Suzuki K, Hasegawa Y, Kitaoka S, Minami Y, Outten FW (2009). J Mol Biol.

[51] Cupp-Vickery JR, Urbina H, Vickery LE (2003). J Mol Biol.

[52] Loiseau L, Ollagnier-de-Choudens S, Nachin L, Fontecave M, Barras F (2003). J Biol Chem.

[53] Outten FW, Wood MJ, Munoz FM, Storz G (2003). J Biol Chem.

[54] Ollagnier-de-Choudens S, Lascoux D, Loiseau L, Barras F, Forest E, Fontecave M (2003). FEBS Lett.

[55] Selbach BP, Pradhan PK, Dos Santos PC (2013). Biochemistry.

[56] Dai Y, Outten FW (2012). FEBS Lett.

[57] Singh H, Dai Y, Outten FW, Busenlehner LS (2013). J Biol Chem.

[58] Selbach B, Earles E, Dos Santos PC (2010). Biochemistry.

[59] Albrecht AG, Netz DJ, Miethke M, Pierik AJ, Burghaus O, Peuckert F, Lill R, Marahiel MA (2010). J Bacteriol.

[60] Riboldi GP, Verli H, Frazzon J (2009). BMC Biochem.

[61] Albrecht AG, Peuckert F, Landmann H, Miethke M, Seubert A, Marahiel MA (2011). FEBS Lett.

[62] Kato S, Mihara H, Kurihara T, Takahashi Y, Tokumoto U, Yoshimura T, Esaki N (2002). Proc Natl Acad Sci USA.

[63] Kornhaber GJ, Snyder D, Moseley HN, Montelione GT (2006). J Biomol NMR.

[64] Vinella D, Brochier-Armanet C, Loiseau L, Talla E, Barras F (2009). PLoS Genet.

[65] Ollagnier-de Choudens S, Nachin L, Sanakis Y, Loiseau L, Barras F, Fontecave M (2003). J Biol Chem.

[66] Ollagnier-de-Choudens S, Sanakis Y, Fontecave M (2004). J Biol Inorg Chem.

[67] Gupta V, Sendra M, Naik SG, Chahal HK, Huynh BH, Outten FW, Fontecave M, de Choudens SO (2009). J Am Chem Soc.

[68] Jensen LT, Culotta VC (2000). Mol Cell Biol.

[69] Wada K, Hasegawa Y, Gong Z, Minami Y, Fukuyama K, Takahashi Y (2005). FEBS Lett.

[70] Tan G, Lu J, Bitoun JP, Huang H, Ding H (2009). Biochem J.

[71] Tirupati B, Vey JL, Drennan CL, Bollinger JM (2004). Biochemistry.

[72] Blauenburg B, Mielcarek A, Altegoer F, Fage CD, Linne U, Bange G, Marahiel MA (2016). PLoS One.

[73] Mihara H, Fujii T, Kato S, Kurihara T, Hata Y, Esaki N (2002). J Biochem (Tokyo).

[74] Goldsmith-Fischman S, Kuzin A, Edstrom WC, Benach J, Shastry R, Xiao R, Acton TB, Honig B, Montelione GT, Hunt JF (2004). J Mol Biol.

[75] Liu G, Li Z, Chiang Y, Acton T, Montelione GT, Murray D, Szyperski T (2005). Protein Sci.

[76] Loiseau L, de Choudens SO, Lascoux D, Forest E, Fontecave M, Barras F (2005). J Biol Chem.

[77] Dai Y, Kim D, Dong G, Busenlehner LS, Frantom PA, Outten FW (2015). Biochemistry.

[78] Kim S, Park S (2013). J Biol Chem.

[79] Kitaoka S, Wada K, Hasegawa Y, Minami Y, Fukuyama K, Takahashi Y (2006). FEBS Lett.

[80] Watanabe S, Kita A, Miki K (2005). J Mol Biol.

[81] Badger J, Sauder JM, Adams JM, Antonysamy S, Bain K, Bergseid MG, Buchanan SG, Buchanan MD, Batiyenko Y, Christopher JA (2005). Proteins.

[82] Tian T, He H, Liu XQ (2014). Biochem Biophys Res Commun.

[83] Cupp-Vickery JR, Silberg JJ, Ta DT, Vickery LE (2004). J Mol Biol.

[84] Morimoto K, Yamashita E, Kondou Y, Lee SJ, Arisaka F, Tsukihara T, Nakai M (2006). J Mol Biol.

[85] Mapolelo DT, Zhang B, Naik SG, Huynh BH, Johnson MK (2012). Biochemistry.

[86] Brancaccio D, Gallo A, Piccioli M, Novellino E, Ciofi-Baffoni S, Banci L (2017). J Am Chem Soc.

[87] Patzer SI, Hantke K (1999). J Bacteriol.

[88] Tanaka N, Kanazawa M, Tonosaki K, Yokoyama N, Kuzuyama T, Takahashi Y (2016). Mol Microbiol.

