# Correction to: Iron–sulfur clusters biogenesis by the SUF machinery: close to the molecular mechanism understanding

**DOI:** 10.1007/s00775-018-1571-7

**Published:** 2018-06-04

**Authors:** J. Pérard, Sandrine Ollagnier de Choudens

**Affiliations:** 1 Laboratoire de Chimie et Biologie des Métaux, Biocat, Université Grenoble Alpes, Grenoble, France; 2 Laboratoire de Chimie et Biologie des Métaux, CNRS, BioCat, UMR 5249, Grenoble, France; 3 CEA-Grenoble, DRF/BIG/CBM, Grenoble, France

## Correction to: JBIC Journal of Biological Inorganic Chemistry 10.1007/s00775-017-1527-3

The article “Iron–sulfur clusters biogenesis by the SUF machinery: close to the molecular mechanism understanding”, written by J. Pérard, Sandrine Ollagnier de Choudens was originally published electronically on the publisher’s internet portal (currently SpringerLink) without open access.

The copyright of the article changed on 29, May to © The Author(s) 2018 and the article is forthwith distributed under the terms of the Creative Commons Attribution 4.0 International License (http://creativecommons.org/licenses/by/4.0/), which permits use, duplication, adaptation, distribution and reproduction in any medium or format, as long as you give appropriate credit to the original author(s) and the source, provide a link to the Creative Commons license and indicate if changes were made.

The original article has been corrected.

